# Hepatitis B virus infections among health professional students in Mwanza city,Tanzania in 2016

**DOI:** 10.1186/s13690-020-00459-2

**Published:** 2020-08-18

**Authors:** Mariam M. Mirambo, Emmanuel Mkumbo, Hadija Selega, Betrand Msemwa, Martha F. Mushi, Vitus Silago, Jeremiah Seni, Stephen E. Mshana, Christa Kasang

**Affiliations:** 1 Department of Microbiology and Immunology, Weill Bugando School of Medicine, Catholic University of Health and Allied sciences, P.O.Box 1464, Mwanza, Tanzania; 2 Institute of Allied Health Sciences, Catholic University of Health and Allied sciences, P.O.Box 1464, Mwanza, Tanzania; 3 Deutsche Lepra- und Tuberkulosehilfe e.V, Raiffeisenstr.3, 97080 Würzburg, Germany

**Keywords:** Anti-HBs, HBsAg, Anti HBV-core, Students, Tanzania

## Abstract

**Background:**

The World Health Organisation (WHO) recommends the vaccination against Hepatitis B virus in all infants and children up to the age of 18 years. In addition, adults in high-risk groups should also be vaccinated. This study investigated the prevalence and factors associated with Hepatitis B Virus (HBV) infections among health professional students in the city of Mwanza, Tanzania in order to provide data that can assist in devising prevention and control strategies in this special group.

**Methods:**

A cross-sectional study involving health professional students of the Catholic University of health and Allied Sciences was conducted between April and July 2016. Hepatitis B surface antigen was detected using rapid antigen test while the anti-hepatitis B surface antibodies(anti-HBs) were quantified using Enzygnost Anti-HBs II assay and anti-HBV core antibodies tested using enzyme immunoassay.

**Results:**

A total of 1211 health professional students with median age of 22 interquartile range (IQR):21–24 years were enrolled. The slighlty majority (57.5%) of these students were males and 475(39.2%) were in clinical practices. Out of 1211 students, 37 (3.1%) were Hepatitis B surface antigen positive. Of 1174 students tested for anti-HBs, 258 (22%) had titres > 10 IU/L indicating HBV immunity. The median anti-HBs titres was 47.7 IU/L(IQR:16–3-113). A total of 230(89.2%) students among those who were positive for anti-HBs were also positive for HBV core antibodies indicating HBV natural infections. Male sex (adjusted odd ratio(AOR):1.77, *p* < 0.000), being married (AOR:1.82, *p* = 0.002) and being in clinical practices (AOR:1.39, *p* = 0.028) independenlty predicted anti-HBs positivity.

**Conclusion:**

A significant proportion of health professional students was naturally immune to Hepatitis B virus. There is a need to measure anti-HBs in order to reduce the cost of unnecessary vaccination especially in the countries with high endemicity of HBV.

## Background

Approximately one third of the global population is infected by Hepatitis B virus(HBV) [1] with about 350–400 million people being chronically infected [2]. High endemicity of HBV is observed in the sub-Saharan Africa and East Asia whereby 5–10% of the adult population is chronically infected. Health professional students are among high risk groups of being infected with the HBV especially during early stage of their clinical practices [3]. Among health care workers the prevalence of chronic infection of 7.4% has been oberved in Tanzania [4] while in Cameroon the prevalence of chronic HBV infection was found to be 11% [5], indicating high endemicity in these countries.

A previous study done at Bugando Medical Centre among health care workers documented the prevalence of HBV natural antibodies of 36.5%, indicating high transmission of HBV infections [4]. However, there is limited information on the magnitude of anti-HBs among health professional students who are also considered as high-risk group that requires vaccination. In addition, the current vaccination practices among children below 5 year of age and among health care workes in Tanzania and in many low-icome countries do not consider the presence of natural antibodies leading to the possibility of unnecessary vaccination in a significant proportion of individuals [6]. Most of studies from low–income countries have estimated the magnitude of chronic HBV infections, with few studies documenting the magnitude of the immunity to HBV [4, 7, 8]. It should be noted that, the use of Hepatitis B surface antigen (HbsAg) does not indicate the true magnitude of HBV infections because the HbsAg indicates only those with chronic/acute infections and not those who have recovered from natural infections. Therefore, in order to combat HBV, the pathogen which has been mentioned in sustainable developmental goals (SDG) ‘Health Goal’, this study was done to estimate the prevalence of HBV infections among health professional students in order to produce data that can be used to formulate strategies to control HBV infections in this high risk population.

## Methods

### Study design, pupulation and area

This was a cross-sectional study which was conducted between April and July 2016 among undergraduate health professional students of the Catholic University of health and Allied Sciences. The Catholic University of Health and Allied sciences is the private University located in the city of Mwanza, Tanzania. It has about 2600 students in various field of health sciences. The study included students from medical laboratory sciences, nursing, medical doctors, radiology and pharmacy programmes.

### Sample size, sampling technique and inclusion criteria

The sample size was estimated by Kish Leslie formula (1965) using the prevalence of 56.7% from previous study which was conducted at Bugando Medical Centre [9] among health care professionals. The minimum sample obtained was 377, however the study enrolled 1211 students. The study included all undergraduate students who consented to participate in the study with no history of HBV vaccination or known positivity of hepatitis B surface antigen. A recruitment centre was set at the University campus for 4 days and serial sampling was used to recruit students as they pass around the recruitment centre. The study did not exclude students on clinical practices because, the aim was establish the magnitude and factors associated with HBV infection. Clinical practices was treated as a factor to confirm what has been documented in previous studies.

### Data collection

A pretested data collection tool was used to collect socio-demographic information and other related information from study participants. Data collected included age, sex, duration on clinical practices, marital status, histroy of unproteced sex etc.

### Hepatitis B surface antigen, anti-hepatitis B surface antibodies(anti-HBs) and anti-hepatitis B core(anti-HBc) assays

About 5-ml of venous blood sample was collected in a plain vacutainer tube(BD, Nairobi, Kenya) under aseptic procedures. Samples were transported to the CUHAS microbiology laboratory whereby blood was centrifuged to obtain sera which was stored in cryovials at -80 °C until analysis. Hepatitis B surface antigen was detected using a qualitative, lateral flow immunochromotography assay test (Accu-Tell Rapid HBsAg Serum/Whole Blood Test, Beijing,101,300,China) following manufacturer instructions. The test has been found to have sensitivity and specificity of > 99.0 and 97.0%, respectively. The anti-HBs were detected using Sandwich ELISA test kit (SIEMENS Enzygnost, Germany) following manufacturer’s instructions, the assay has sensitivity and specificity of more than 95% while anti-HBc antibodies were detected using enzyme immunoassay (Elite Medical Company, India) as per manufacturer instructions, this assay has sensitivity and specificity of more than 95%.

### Data analysis

Continuous variables were summarized as median with inter-quartile range while categorical variables were summarized as proportions. Univariable analysis was done followed by multivariable logistic regression analysis to determine the predictors of HBsAg positivity and the presence of anti-HBs. The titre of anti-HBs of ≥10 IU/L was considered as the presence of natural immunity to HBV. The medians were compared using the Wilcoxon rank-sum Mann Whitney test. Factors with a *p*-value of < 0.2 on univariable analysis were subjected into multivariable logistic regression analysis. All factors with *p*-values of < 0.05 at 95% confidence interval were considered statistically significant. Age was not subjected on the multivariable analysis due to its collinearity with being in clinical practices.

## Results

### Sociodemographic characteristics of the study participants

A total of 1211 students were enrolled, representing 46% of the Catholic University of Health and Allied Sciences students’ population in 2016. The median age was 22[IQR:21–25] years with the slightly majority 57.5% (696/1211) of the students being male. Regarding marital status, the majority of the participants 86.1% (1043/1211) were single. Out of 1211 students, 475(39%) had started clinical practices(Table 1). None of these students had received HepB vaccination before.

**Table 1:** Sociodemographic and clinical characteristics of unvaccinated health professional students at Catholic University of Health and Allied Sciences, April to July 2016, Mwanza Tanzania

**Participant Characteristics**	**1174 students tested anti-HBs**		**1211 students tested anti-HBs**	
	**Number**	**Percent[%]/Median [IQR]**	**Number**	**Percent[%]/Median [IQR]**
**Age IQR [years]**	1174	22 [21–24] years	1211	22(21–25) years
**Sex**				
Male	672	57.24	696	57.5
Female	502	42.76	515	42.5
**Marital Status**				
Single	1014	86.37	1025	84.6
Married	160	13.63	186	15.4
**Clinical Practise**				
Yes	458	39.01	475	39
No	716	60.99	736	61

### Prevalence of hepatitis B surface antigen and anti-HBsAg

Out of 1211 students tested for hepatits B surface antigen(HbsAg), 37(3.1, 95%CI: 2.1–4.0) were HbsAg positive. All students (1174) who were HBsAg negative were tested for anti-HbsAg, of these, 258(22, 95%CI: 19.6–24.4) had titres > 10 IU/L, indicating immunity to HBV. Low, moderate and high anti-HBs titers were observed in 69.4%(179/258), 25.2% (65/258) and 5.4% (14/258) students, respectively (Table 2). The median anti-HBs titres was 47.7 IU/L(IQR:16–3-113). Out of 258 tested positive for anti-HBs, 230(89.2%) were positive for anti-HBc indicating natural infections.

**Table 2:** Prevalence of Hebatitis B surface antigen and Anti-HbsAg antibodies among health professional students from CUHAS, April to July 2016, Mwanza, Tanzania

**Variable**	**n**	**%**
**Hepatitis B surface antigen (*N*** **= 1211)**	37	3.1
**HbsAb Status (*N*** **= 1174)**		
Positive [≥10 IU/L]	258	22.0
Negative [< 10 IU/L]	916	78.0
**Anti-HBs titres (258)**		
High [> 200 IU/L]	14	5.4
Moderate [100-200 IU/L]	65	25.2
Low [10-100 IU/L]	179	69.4

### Factors associated with HBV infections among health professional students

The median age of partcipants with HBV immunity was significantly higher than those with no immunity (23 IQR;22–26 vs. 22 IQR; 21–24, *P* < 0.001). Out of 502 females, 81(16.1%) were positive for anti-HBs compared to 177(26.3%) of 672 males, *p* < 0.001. Being in clinical practices was found to be associated with the presence of anti-HBs (27.2% vs. 18.7%, *P* = 0.001). By multivariable logistic regression analysis; male sex (OR:1.77, 95% CI:1.31–2.38), being married (OR:1.82, 95% CI:1.25–2.64) and being in clinical practices (OR:1.39, 95% CI:1.03–1.86) were found to predict anti-HBs positivity (Table 3). None of the factors studied (sex, age, clinical practices, marital status and practice unprotected sex) were found to be associated with HBsAg positivity.

**Table 3:** Factors associated with anti-HBsAg among 1174 unvaccinated health professional CUHAS students April to July 2016, Mwanza, Tanzania

**Variable**	**Anti-HBsAg**	**Univariate**	**Mutivariable**
		**OR[95%CI]**	**OR[95% CI]**
**Age (yrs)**	23 IQR[22–26]	1.05 [1.03–1.07]	
**Sex**			
Female	81 [16.14]	1	
Male	177 [26.34]	1.85 [1.38–2.49]	1.77[1.31–2.38]
**Marital Status**			
Single	205 [20.22]	1	
Married	53 [33.13]	1.95 [1.35–2.81]	1.82[1.25–2.64]
**Clinical Practices**			
No	134 [18.72]	1	
Yes	124 [27.07]	1.61 [1.22–2.13]	1.39 [1.03–1.86]
**Unprotected Sex**			
No	173 [20.50]	1	
Yes	85 [25.76]	1.34 [0.99–1.81]	1.06 [0.77–1.46]

## Discussion

The overall prevalence of HBsAg was 3.1% among CUHAS health professional students. The observed prevalence was significantly lower than what was reported in previous studies in Makerere Uganda (18%) and in Kenyatta National hospital(11%) among medical students [10, 11]. The low prevalence of HBsAg could indicate high natural immunity in this population or high vaccine coverage, however this was not the case in this population because none of student had received HBV vaccination. In Tanzania, at the time of data collection the HBV immunization programme for children below 5 years of age was just 6 years old, therefore it was unlikely that these students were immunized before.

In this study, the overall prevalence of anti-HBs was found to be 22% with 89.2% of these students being positivite for anti-HBc indicating that, the majority of anti-HBs were due to natural infections. This was comparable with a previous study in South Africa [12] which reported prevalence of 18.8% among health care workers. However, when compared to the prevalence of immunity among the students who were in clinical practices the oberved prevalence of HBV immunty in the current study was significantly high indicating high transmission of HBV in this setting. The observation was further supported by the previous study in the same setting which observed the prevalence of natural immunity of 36.5% among health care workers. However, the observed immunity in the current study was comparable to 25.3% observed among febrile patients in two districts hospitals in Tanzania [13].

The difference between this study and that of South Africa could be explained by the fact that in South Africa the HBV vaccination among health care workers was introduced earlier than in Tanzania [5, 14]. South Africa and Tanzania has similar endemicity status as per World Health Organization (WHO), this further supports the similarity in the general prevalence of HBV natural immunity.

In comparison to the previous study in China which reported the prevalence of 13%, the prevalence reported in this study is indeed high [15]. This could be due to the fact that in China HBV vaccination campaign has been there for many years [16].

None of the factors studied was found to predict HbsAg positivity, however male sex, being married and being in clinical practices were found to predict anti-HBs positivity in the present study. Previous studies [4, 17] showed the association between HBV infection and the duration of employment/clinical practices, emphasizing the need to vaccinate health professional students to prevent subsequent infections.

In this study male sex was significantly associated with HBV immunity. This could be explained by the fact that in Tanzania boys tend to be more involved in high risk behaviours than girls [18]. Further multi -centre studies to asess the association between HBV infections and gender among students are recommended so that tartegted health education can be given. Furthermore, being married was found to predict HBV natural infections in this study confriming what was oberved in previous studies [19, 20].

It should be noted that the 96 ELISA kit for for screening of the anti-HBs costs about 500USD(i.e. about 6USD per test) compared to the cost of single dose HBV vaccine of about 12.3USD [19]. Therefore, screening for anti-HBs should be recommnded in the countries with HBV high endemicity in order to avoid unnecessary costly vaccination. A previous study in China concluded that HepB vaccination campaign with screening provided more greater value than a vaccination without screening vaccination [19]. Furthermore, there is a need to invest on point of care anti-HBs assays that can be used in developing countries to asses the level of anti-HBs before vaccination.

In this study non significant association was observed between unprotected sexual intercourse and HBV infections. This could be contributed to the validity of the self-response regarding the issues of unprotected sexual intercourse. Self reports on the issue of sexual behaviours can be affected by both cognitive and situational factors in varying degrees [21]. As major limitation no attempt was made to validate the information given from these students due the design of this study.

## Conclusion

A significant proportion of students undertaking health sciences courses were found to be naturally immune to HBV. The immunity was significanlty predicted by male sex, being in clinical practices and being married. This calls for the need to emphasize the screening for the presence of anti-HBs so that the vaccine can be given to those who are eligible to avoid waste vaccinations among those with natural immunity, this will ensure costeffective implementation of of HBV vaccination programme. If the implementation of universal screening for anti-HBs is considered as difficult option, it is recommended that in the Higher learning institutions for health in developing countries, married-male students in their clinical practices should mandatory be screened for anti-HBs before provision of HBV vaccination. This will significantly minimize the cost of HBV vaccination to the institutions and respective individual students.

## Data Availability

The data is available upon request and the request should be made to the Director of research and Innovation Catholic University of Health and allied Sciences.

## Abbreviations

CUHAS: Catholic University of Health and Allied Sciences
CI: Confidence interval
ELISA: Enzyme-linked immunosorbent assay
HBV: Hepatitis B Virus
HBs: Hebatitis B surface antitigen
HBc: Hebatitis B core antigen
IU: International Unit
IQR: Interquartile range
OR: Odd ratio

## References

[1] Hwang EW, Cheung R (2011). Global epidemiology of hepatitis B virus (HBV) infection. N Am J Med Sci.

[2] McMahon BJ (2004). Epidemiology and natural history of hepatitis B. Seminars in liver disease.

[3] Wang H, Fennie K, He G, Burgess J, Williams AB (2003). A training programme for prevention of occupational exposure to bloodborne pathogens: impact on knowledge, behaviour and incidence of needle stick injuries among student nurses in Changsha, People's Republic of China. J Adv Nurs.

[4] Mueller A, Stoetter L, Kalluvya S, Stich A, Majinge C, Weissbrich B, Kasang C (2015). Prevalence of hepatitis B virus infection among health care workers in a tertiary hospital in Tanzania. BMC Infect Dis.

[5] Tatsilong HOP, Noubiap JJN, Nansseu JRN, Aminde LN, Bigna JJR, Ndze VN, Moyou RS (2016). Hepatitis B infection awareness, vaccine perceptions and uptake, and serological profile of a group of health care workers in Yaoundé, Cameroon. BMC Public Health.

[6] Organization WH (2019). Hepatitis B vaccines: WHO position paper, July 2017–recommendations. Vaccine.

[7] Tandon B, Irshad M, Raju M, Mathur G, Rao M (1991). Prevalence of HBsAg & anti-HBs in children & strategy suggested for immunisation in India. Indian J Med Res.

[8] Soběslavský O (1980). Prevalence of markers of hepatitis B virus infection in various countries: a WHO collaborative study. Bull World Health Organ.

[9] Mueller A, Stoetter L, Kalluvya S, Stich A, Majinge C, Weissbrich B, Kasang C (2015). Prevalence of hepatitis B virus infection among health care workers in a tertiary hospital in Tanzania. BMC Infect Dis.

[10] Pido B, Kagimu M (2005). Prevalence of hepatitis B virus (HBV) infection among Makerere University medical students. Afr Health Sci.

[11] Lule G, Okoth F, Ogutu E, Mwai S (1989). HBV markers HBsAg, HBSAb, HBCAb in 160 medical students at Kenyatta National Hospital. East Afr Med J.

[12] Sondlane TH, Mawela L, Razwiedani LL, Selabe SG, Lebelo RL, Rakgole JN, Mphahlele MJ, Dochez C, De Schryver A, Burnett RJ (2016). High prevalence of active and occult hepatitis B virus infections in healthcare workers from two provinces of South Africa. Vaccine.

[13] Meschi S, Schepisi MS, Nicastri E, Bevilacqua N, Castilletti C, Sciarrone MR, Paglia MG, Fumakule R, Mohamed J, Kitwa A (2010). The prevalence of antibodies to human herpesvirus 8 and hepatitis B virus in patients in two hospitals in Tanzania. J Med Virol.

[14] Burnett RJ, Kramvis A, Dochez C, Meheus A (2012). An update after 16 years of hepatitis B vaccination in South Africa. Vaccine.

[15] Liao X-Y, Zhou Z-Z, Wei F-B, Qin H-N, Ling Y, Li R-C, Li Y-P, Nong Y, Sun K-X, Li J (2014). Seroprevalence of hepatitis B and immune response to hepatitis B vaccination in Chinese college students mainly from the rural areas of western China and born before HBV vaccination integrated into expanded program of immunization. Human Vaccines Immunotherapeutics.

[16] Liang X, Bi S, Yang W, Wang L, Cui G, Cui F, Zhang Y, Liu J, Gong X, Chen Y (2013). Reprint of: epidemiological serosurvey of hepatitis B in China—declining HBV prevalence due to hepatitis B vaccination. Vaccine.

[17] Techasathit W, Ratanasuwan W, Sonjai A, Sangsiriwut K, Anekthananon T, Suwanagool S (2005). Vaccination against hepatitis B virus: are Thai medical students sufficiently protected. J Med Assoc Thail.

[18] Maswanya E, Moji K, Horiguchi I, Nagata K, Aoyagi K, Honda S, Takemoto T (1999). Knowledge, risk perception of AIDS and reported sexual behaviour among students in secondary schools and colleges in Tanzania. Health Educ Res.

[19] Tahan V, Karaca C, Yildirim B, Bozbas A, Ozaras R, Demir K, Avsar E, Mert A, Besisik F, Kaymakoglu S (2005). Sexual transmission of HCV between spouses. Am J Gastroenterol.

[20] Inaba N, Ohkawa R, Matsuura A, Kudoh J, Takamizawa H (1979). Sexual transmission of hepatitis B surface antigen. Infection of husbands by HBsAg carrier-state wives. Sex Transm Infect.

[21] Brener ND, Billy JO, Grady WR (2003). Assessment of factors affecting the validity of self-reported health-risk behavior among adolescents: evidence from the scientific literature. J Adolesc Health.

